# *In vivo* assessment of the antiproliferative properties of interferon-alpha during immunotherapy: Ki-67 (MIB-1) in patients with metastatic renal cell carcinoma

**DOI:** 10.1038/sj.bjc.6601587

**Published:** 2004-02-03

**Authors:** F Donskov, N Marcussen, M Hokland, R Fisker, H H T Madsen, H von der Maase

**Affiliations:** 1 Department of Oncology, Aarhus University Hospital, Denmark; 2 Institute of Pathology, Aarhus University Hospital, Denmark; 3 Institute of Medical Microbiology and Immunology, University of Aarhus, Denmark; 4 Department of Radiology, Aarhus University Hospital, Denmark

**Keywords:** renal cell carcinoma, Ki-67, MIB-1, interferon-*α*, interleukin-2, prognostic factors

## Abstract

The aim of the present study was to investigate the *in vivo* antiproliferative effect of interferon alpha (IFN-*α*) in patients with metastatic renal cell carcinoma (mRCC). Core needle biopsies of metastatic and/or the primary kidney cancer were obtained before interleukin-2 (IL-2)- and IFN-*α*-based immunotherapy in 34 patients and repeated after 5 weeks in 25 patients. Tumour proliferation was assessed by use of the anti-Ki-67 antibody MIB-1 and evaluated in multiple, random systematic sampled fields of vision. Ki-67 labelling index (LI) at baseline was median 13.6% (range 1.2–85.0) and median 10.6% (range 1.3–48.6%) at week 5 with a median overall decline of 15.2% (range −95 to +258%) from baseline to week 5. There was no difference between responding and nonresponding patients. Ki-67 LI at week 5 was significantly correlated to survival. Thus, median survival of patients with Ki-67 LI ⩽10.6% at week 5 was 25.1 months compared to 11.5 months for patients with Ki-67 LI >10.6% (*P*=0.016). Baseline or change in Ki-67 LI did not correlate to survival. These data suggest that IFN-*α in vivo* has only modest effect on tumour proliferation in patients with mRCC. Tumour Ki-67 (MIB-1) reactivity after 1 month of immunotherapy appears to be a significant predictor of patient survival.

Metastatic renal cell carcinoma (mRCC) is refractory to conventional therapies including radiation, hormones and chemotherapy (Motzer and Russo, 2000). Patients with untreated mRCC have a poor prognosis with a median survival of only 8 months (Haas *et al*, 1993). However, interleukin-2 (IL-2)- and interferon-*α* (IFN-*α*)-based immunotherapy can induce durable tumour regression in 5–10% of patients with mRCC (Minasian *et al*, 1993; Rosenberg, 2001).

Understanding of the mechanisms by which IL-2 and IFN-*α* mediate their antineoplastic actions is incomplete. Whereas cellular immune effector mechanisms are considered to be the most important mediators of IL-2 antitumour activity (Rubin *et al*, 1989; Donskov *et al*, 2002a), IFN-*α* in addition (Kosmidis *et al*, 1992; Tsavaris *et al*, 1996; Donskov *et al*, 2002a) is considered to exert antiproliferative (Grander *et al*, 1997) and differentiation-inducing effects on the tumour cells (Pfeffer *et al*, 1998). *In vitro*, a direct antiproliferative effect on renal tumour cells has been demonstrated for IFN-*α* (Nanus *et al*, 1990), whereas IL-2 has no direct impact on cancer cells (Rosenberg, 2001). However, despite 45 years since the discovery of IFN (Pfeffer *et al*, 1998), only one study has evaluated the antiproliferative effect of IFN *in vivo* in patients with mRCC (Yoshino *et al*, 2000). In that study, however, only baseline tumours were analysed and no correlation to objective response or survival was found (Yoshino *et al*, 2000).

A commonly used marker of tumour proliferation is Ki-67 (MIB-1), a monoclonal antibody recognising a nuclear protein expressed in all active phases of the cell cycle (G_1_, S, G_2_ and M) but absent in quiescent cells (G_0_) (Cattoretti *et al*, 1992). In nephrectomy specimens of nonmetastatic RCC (Tannapfel *et al*, 1996; Aaltomaa *et al*, 1997; Kirkali *et al*, 2001; Rioux-Leclercq *et al*, 2001) and mRCC (Rioux-Leclercq *et al*, 2001), Ki-67 (MIB-1) labelling index (LI) has been demonstrated as an independent parameter of unfavourable prognosis.

To assess the *in vivo* antiproliferative properties of IFN-*α* during treatment, we have monitored Ki-67 (MIB-1) LI in repeated core needle tumour biopsies obtained at baseline and at week 5 of IL-2- and IFN-*α*-based immunotherapy in patients with mRCC and correlated the findings with objective response and survival.

## MATERIALS AND METHODS

### Patients and samples

A total of 49 consecutive single-institution patients with mRCC were treated on an outpatient basis from February 1999 to August 2000 at the Department of Oncology, Aarhus University Hospital. Of these, 26 were enrolled in a Scandinavian, multicentre prospective phase II trial of s.c. IL-2, IFN-*α* and histamine dihydrochloride (Donskov *et al*, 2002b). The treatment plan consisted of one priming-week of daily IFN-*α* and up to nine treatment cycles of 4 weeks with IFN-*α* (human leucocyte IFN-*α*, Interferon Alfanative®, BioNative, Sweden, 3.0 MIU as a fixed dose, s.c. once daily, 7 days per week throughout the study), IL-2 (Aldesleukin, rIL-2, Proleukin®, Chiron, The Netherlands, 2.4 MIU m^−2^, s.c., two times daily 5 days per week, weeks 1 and 2 every cycle) and histamine (Maxim Pharmaceuticals Inc., San Diego, CA, USA, 1.0 mg in 1.0 ml by 20 min slow s.c. injections, two times daily, 5 days per week throughout the study).

The subsequent 23 patients were treated with the same schedule as the phase II trial, but without histamine (Donskov *et al*, submitted for publication). Moreover, instead of leucocyte IFN, which was used because of the tradition in Sweden, recombinant IFN*α*-2b (Introna®, Schering-Plough, Denmark) was applied by the same dose and schedule.

Histamine did not add efficacy with respect to response, time to progression or survival, and did not influence levels of intratumoral or blood leucocyte numbers, zeta chain expression or cytotoxicity (Donskov *et al*, 2003). Thus, the present study was based on the pooled data from these two treatment groups.

Of the 49 patients, 45 gave written informed consent for sequential core needle biopsies. Two patients did not complete one course of therapy because of toxicity and were not evaluable for objective response. These two patients were excluded from all further analyses. Two patients were excluded from the present study using core needle biopsies as they had only fine needle biopsies performed. Three patients had no biopsies performed for safety reasons because tumours were not accessible for core needle biopsies (metastases located in lung, bone, mediastinal lymph node or retrocrural lymph node close to aorta). No complete responding patients had accessible tumours for core needle biopsies. Thus, a total of 38 patients were included in the present study with sequential core needle tumour biopsies. Core needle biopsies (18G cutting needle) were collected by standard ultrasound-guided procedures (Jennings *et al*, 1989) at baseline and after 1 month of immunotherapy on day 1 in the fifth treatment week. On-treatment biopsy was obtained from the same tumour as baseline. A total of 76 core needle biopsies in 38 patients were performed at different tumour sites (kidney, *n*=43; abdominal soft tissue, *n*=10; liver, *n*=8; pleura, *n*=4; muscle, *n*=4; kidney bed, *n*=3; subcutis, *n*=2; and lymph node, *n*=2). There were four nonevaluable patients for the analyses, two because of insufficient tumour tissue in the biopsies and two because of necrosis in all biopsies at both biopsy time points. Eight patients had only a baseline biopsy performed (withdrawal of consent). Of the 76 biopsies, 13 (17%) were excluded because of necrosis. Thus, at baseline and after 1 month of immunotherapy, 34 and 25 patients, respectively, had evaluable biopsies. Patient characteristics for the 34 patients are given in Table 1

**Table 1:** Baseline patient characteristics (*n*=34)

Median age, years (range)	56 19–74
Sex	
Male	26 76%
Female	8 24%
Karnofsky performance status	
100	11 32%
90	11 32%
80	6 18%
70	6 18%
Prior therapy	
Nephrectomy	12 35%
Excision of metastatic lesions	6 18%
None	19 56%
Number of disease sites	
1	1 3%
2	10 29%
3	12 35%
4 or more	11 32%
Most common sites of disease	
Primary kidney tumour	22 65%
Local recurrence kidney bed	7 21%
Lung/pleura	20 59%
Lung metastasis alone	0 0%
Lymph node	17 50%
Liver	10 29%
Bone	13 38%
Soft tissue	10 29%

. Distribution of prognostic factors according to Memorial Sloan Kettering Cancer Center (MSKCC) (Motzer *et al*, 1999) and UCLA Integrated Staging System (Zisman *et al*, 2001) are given in Table 2a and b

**Table 2:** Distribution of prognostic factors

	**Consecutive patients**		**Evaluable patients**			**Baseline patients**			**Week-5 patients**		
**Prognostic group**	***n*=49**	**%**	***n*=46**	**%**	***P***	***n*=34**	**%**	***P***	***n*=25**	**%**	***P***
*(a) MSKCC*^a^											
Favourable	3	6%	3	7%	0.66	2	6%	0.70	2	8%	0.59
Intermediate	25	51%	24	52%		16	47%		14	56%	
Poor	21	43%	19	41%		16	47%		9	36%	

*(b) UISS*^b^											
	**Baseline patients**		**Week-5 patients**								
	***n*=34**	**%**	***n*=25**	**%**	***P***						
UISS 3	6	18%	5	20%	1.00						
UISS 4	27	79%	19	76%							
UISS 5	1	3%	1	4%							

*P*=Fisher's exact test.

a Motzer *et al* (1999). Risk factors: Karnofsky PS <80, LDH ⩾1.5 times upper limit of normal, haemoglobin <lower limit of normal, corrected s-Ca >10 mg dl^−1^ or ion-Ca >upper limit of normal and no prior nephrectomy. No risk factors=favourable prognosis, 1–2 risk factors=intermediate prognosis and >2 risk factors=poor prognosis.

b UCLA Integrated Staging System (Zisman *et al*, 2001), based on TNM stage, Fuhrman grade and performance status.

.

According to the Union Internationale Contre le Cancer (UICC) and the American Joint Committee on Cancer (AJCC) 1997 classification (Reuter and Presti, 2000), all patients had conventional (clear cell) RCC except two, who had papillary RCC and collecting duct RCC. These two patients achieved stable disease and progressive disease, respectively. All patients had stage IV disease, according to the TNM classification (Guinan *et al*, 1997).

### Immunohistochemistry

Sections (2 *μ*m) of formalin-fixed, paraffin-embedded biopsies were mounted on ChemMate slides (cat. no. S2024 DAKO, Denmark), dried 1 h at 60°C, deparaffinised and rehydrated. After endogenous peroxidase blocking (0.5% hydrogen peroxidase in water for 30 min), antigens were retrieved by microwave oven heating (3 × 5 min at 850 W in Tris/EGTA retrieval buffer (pH 9.0)). The tissue sections were incubated for 1 h with the Ki-67 (MIB-1) antibody diluted 1 : 100 (cat. no. IM 0505 Immunotech). As second layer, EnVision (cat. no. K4000, DAKO) was used for 30 min of incubation. Staining was visualised with diaminobenzidine tetrahydrochloride solution, counterstained in Mayer's haematoxylin and mounted with Aqutex (64912-50, KEBO-lab, Denmark). All staining was performed in a TechMate automate machine (DAKO). As positive controls, a normal lymph node and several non-mRCC tumours were used. As negative controls, substitution of primary antibody with PBS and isotype IgG1 (cat. no. 33811A, Pharmingen, Denmark) 1 : 50 was used.

### Immunohistochemical evaluation

Stereologic counting of Ki-67 was performed using a light microscope equipped with a CAST-grid software package (version 2.0, Olympus, Denmark) for manual interactive counting on a computer screen, as previously described in detail (Gundersen *et al*, 1988; Jensen *et al*, 1998). In brief, the stereologic estimates were based on random and systematic sampling of counting fields (4989 *μ*m^2^ each) in the tumour tissue. A motorised stage, controlled by the computer, made it possible to sample the first counting field at random and then to move systematically throughout the tumour section. Using a × 40 objective, a median number of 30 fields (range 12–30) and a median number of 444 tumour cells (range 49–949) were counted. The entire core needle biopsy was assessed. Areas of necrosis were avoided. Tumour cells were considered positive for Ki-67, if there was any staining of the nucleoplasm or nucleoli, regardless of staining intensity. Proliferating tumour cells were distinguished from proliferating intratumoral immune cells on the basis of morphology and size. In case of doubt, support from CD3 immunostaining (Donskov *et al*, 2002a) was obtained. The labelling index of Ki-67 (Ki-67 LI) was defined as the percentage of Ki-67-labelled tumour cells to the total number of tumour cells counted. Staining was analysed blinded by one observer (FD). For testing the reproducibility, sections were ranked according to their number of proliferative cells and every sixth case was selected and counted blinded by a senior histopathologist (NM). A high level of reproducibility (Spearman's ρ=0.90, *P*=0.0001) was found.

### Statistics

Overall survival was measured from first day of treatment until death or last follow-up evaluation. The relationship between assessed parameters and objective response was evaluated using the nonparametric Mann–Whitney U test. The significance of changes from baseline to week 5 was assessed using the Wilcoxon's signed rank test for paired samples. The relationship between assessed parameters and survival was evaluated using the method of Kaplan–Meier and the log-rank test. Dichotomy of the patients was carried out at the median value for the evaluated parameter. All tests were two-sided and the significance level was 0.05. The median follow-up period was 44.7 months (range 33.3–50.4 months). Data were updated in June 2003. Statistical analyses were performed using SPSS v10.0.

## RESULTS

### Clinical treatment results

A total of 34 patients treated with low dose IL-2, IFN-*α* with or without histamine were evaluable for consecutive tumour biopsies. Of these, six patients achieved partial remission (PR), 11 patients achieved stable disease (SD) and 17 patients had progressive disease (PD). Median survival was 13.3 months (range 1.3–50.4+ months). Four patients (three with PR and one with SD) had no evidence of disease (NED) and were alive at 33+, 40+, 47+ and 50+ months, respectively, after immunotherapy followed by subsequent resection of residual tumour. At the time of analysis, 28 patients had died, giving a censoring rate of 17.7%.

### Correlation between tumour nuclear Ki-67 (MIB-1) staining and objective response

**Figure 1:**
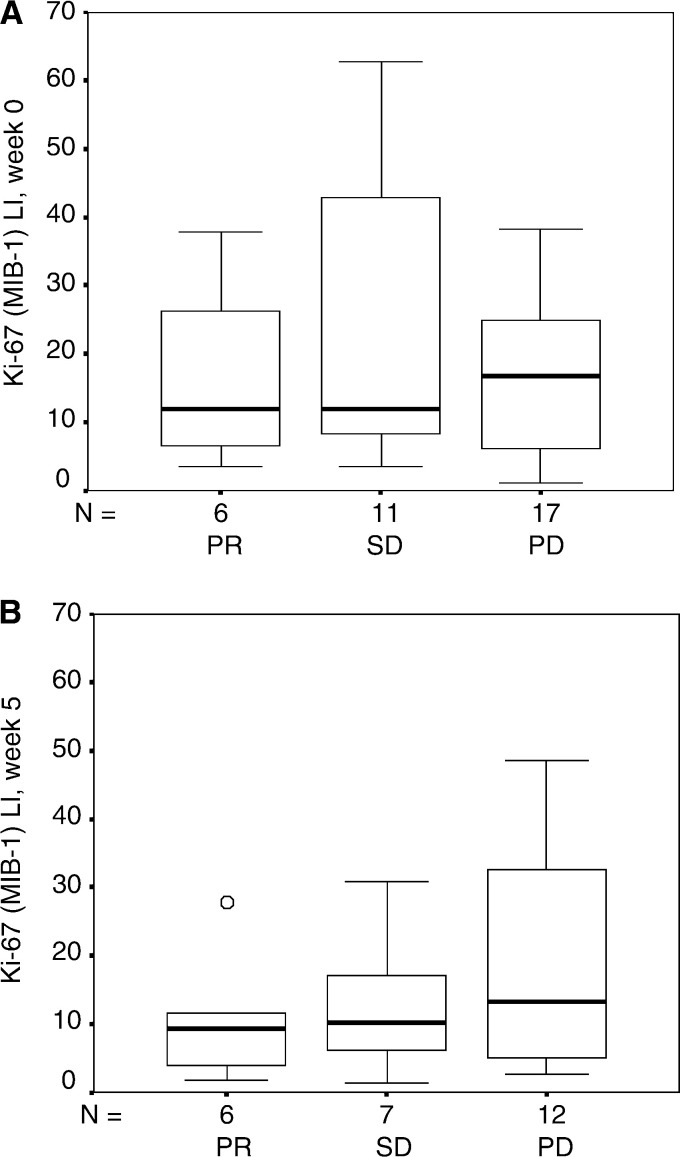
Tumour cell proliferation marker Ki-67 (MIB-1) LI in patients with mRCC (**A**) before and (**B**) after 1 month of interleukin-2- and interferon-*α*-based immunotherapy for patients obtaining partial response (PR), stable disease (SD) and progressive disease (PD). The box plots represent the median, the 25th and the 75th percentiles, respectively. The error bars represent the 10th and the 90th percentiles, respectively.

Baseline and on-treatment tumour proliferation defined by Ki-67 immunohistochemistry staining were evaluated and correlated to objective response. The Ki-67 (MIB-1) LI was median 13.6% (range 1.2–85.0%) at baseline and median 10.6% (range 1.3–48.6%) after 1 month of therapy (Figure 1

). When Ki-67 LI of responding (PR) and nonresponding patients (SD, PD) at baseline and after 1 month of therapy was compared, no significant differences were noted (*P*=0.7 and 0.4, respectively). Also when Ki-67 LI of PD patients and non-PD patients (PR, SD) at baseline and after 1 month of therapy was compared, no significant differences were noted (*P*=0.7 and 0.3, respectively). However, patients who obtained no evidence of disease (NED, *n*=4) had significantly lower Ki-67 LI (*P*=0.022) than non-NED patients (*n*=21) at week 5, whereas no difference was found (*P*=0.4) at baseline.

**Figure 2:**
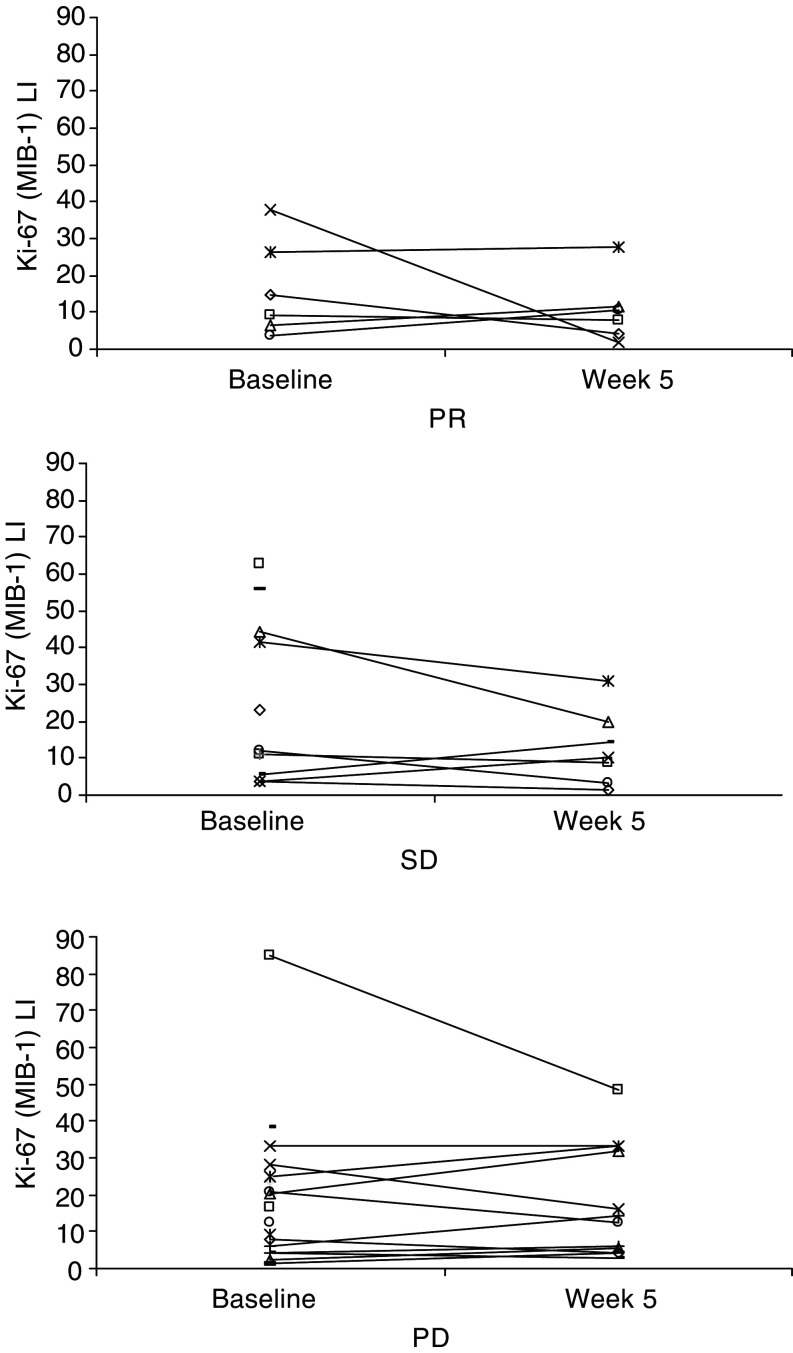
Change in Ki-67 (MIB-1) LI in per cent at baseline and after 1 month of interleukin-2- and interferon-*α*-based immunotherapy for patients obtaining partial response (PR), stable disease (SD) and progressive disease (PD). The data points represent the Ki-67 (MIB-1) scores for individual patients, a total of 34 patients at baseline and 25 patients after 1 month of immunotherapy.

A median overall decline of 15.2% (range –95 to +258%) in Ki-67 tumour expression from baseline to week 5 was observed (*P*=0.39) (Figure 2

). When correlated to objective response, no significant difference (*P*=0.8) was found.

### Correlation between tumour nuclear Ki-67 (MIB-1) staining and survival

**Figure 3:**
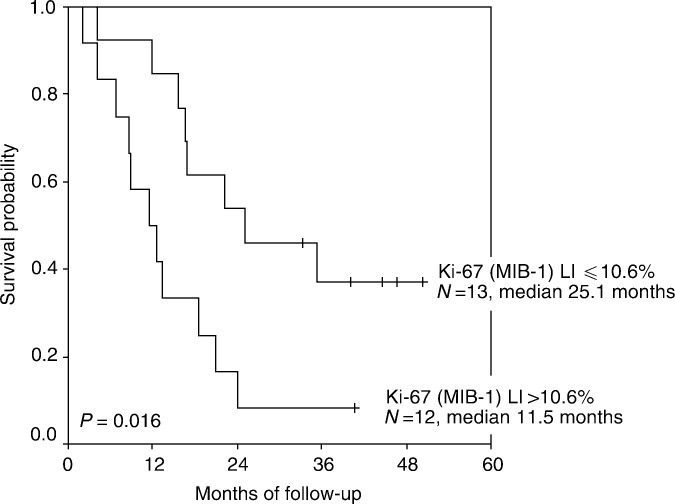
Kaplan–Meier survival estimate demonstrating Ki-67 (MIB-1) LI in tumour after 1 month of interleukin-2- and interferon-*α-* based immunotherapy as a negative prognostic factor for survival in patients with mRCC. Tick marks represent six censored patients.

We also studied the influence of tumour proliferation on survival. Ki-67 (MIB-1) LI at week 5 was statistically significantly associated to survival (Figure 3

). Median survival of patients with tumour cell proliferation ⩽10.6% at week 5 was 25.1 months compared to 11.5 months for patients with tumour cell proliferation >10.6% (*P*=0.016). Baseline or change in Ki-67 (MIB-1) LI was not correlated to survival (*P*=0.25 and 0.65, respectively).

## DISCUSSION

This is to our knowledge the first *in vivo* serial assessment of the antiproliferative properties of IFN-*α* during immunotherapy in patients with mRCC. We observed only modest reduction in the number of proliferating tumour cells induced by IFN-*α-* and IL-2-based immunotherapy. Tumour Ki-67 (MIB-1) expression after 1 month of immunotherapy appeared to be a predictive marker of survival, whereas at baseline, this marker failed as a marker of unfavourable prognosis. The implication is that the proliferative activity assessed after 1 month of immunotherapy has a greater value as prognostic factor of survival as compared with baseline assessment. One possible explanation might be that whereas the majority of destruction of sensitive tumour targets appear within the first courses of immunotherapy (Lindsey *et al*, 2000), resulting in immunological ‘shaping’ or ‘sculpting’ of the tumours (Khong and Restifo, 2002), the tumour after 1 month of immunotherapy is less sensitive to immunotherapy and, thus, assessment of tumour proliferation at this time has a greater value as a prognostic factor for survival.

The general concept that aggressive tumours with elevated baseline proliferative activity have a poor outcome in spite of the treatment administered is not supported by our findings. We also failed to demonstrate that long-term surviving patients after immunotherapy treatment only had tumours with low baseline proliferative activity, as has been reported in metastatic melanoma (Vlaykova *et al*, 1999). In other words, the fate of a patient with mRCC prior to IL-2- and IFN-*α*-based immunotherapy cannot be determined by measuring baseline Ki-67 (MIB-1) LI.

Only 25 of 49 (51%) treated patients had evaluable biopsies after 1 month of treatment. Therefore, to make meaningful comparisons, a larger number of subjects are required. However, it should be noted that patients at week 5 were randomly selected from, or were at least representative of, a larger population, based on well-known prognostic factors of MSKCC (Motzer *et al*, 1999) and UISS (Zisman *et al*, 2001) (Table 2a and b).

*In vitro*, a direct antiproliferative effect on renal tumour cells has been demonstrated for IFN-*α* (Nanus *et al*, 1990). IL-2 has no direct impact on cancer cells, which can grow unimpeded *in vitro* in high concentrations of IL-2 (Rosenberg, 2001). *In vitro*, histamine inhibits oxygen radical formation in monocytes and thus reverses oxidative inhibition of T cells and NK cells (Hellstrand, 2002). No direct proliferative effect on renal tumour cells has been demonstrated for histamine *in vitro*. However, by *in vivo* administration of histamine to nonrenal tumour-bearing rodents, both enhancement and suppression of tumour growth have been reported (Hellstrand *et al*, 2000). This discrepancy may be related to differential effects on the immune system rather than proliferative effects on the tumour cells (Hellstrand *et al*, 1990). Thus, despite patients in the present study received combination immunotherapy and not single-agent IFN-*α*, it seems reasonable to conclude that the observed antiproliferative effects are caused by IFN-*α*.

We demonstrated only modest reduction in the number of proliferating tumour cells induced by IFN-*α*. This finding is in accordance with the results of two recent randomised phase III trials in mRCC demonstrating a significant but only modest effect of IFN-*α* (Medical Research Council Renal Cancer Collaborators, 1999; Pyrhonen *et al*, 1999). Thus, a British study with a total of 335 patients randomised to subcutaneous IFN-*α* or oral medroxyprogesterone acetate (MPA) (Medical Research Council Renal Cancer Collaborators, 1999) demonstrated a significant absolute improvement in 1-year survival of 12% (43 *vs* 31%) and a significant improvement in median survival of 2.5 months (8.5 *vs* 6.0 months) for IFN-*α*-treated patients compared to MPA-treated patients. Likewise, a Finnish study with a total of 160 patients randomised to IFN-*α* plus vinblastine or vinblastine alone (Pyrhonen *et al*, 1999) demonstrated a significant absolute improvement in 1-year survival of 18% (56 *vs* 38%) and a significantly prolonged median survival of 7.4 months (67.6 *vs* 37.8 weeks) for patients receiving both drugs. So although IFN-*α* has reproducible antitumour effects in mRCC, these data demonstrate the modest effect in mRCC and emphasise that novel treatment strategies and identification of new agents with better antitumour activity remain a high priority.

Despite 45 years since the discovery of IFN (Pfeffer *et al*, 1998) and two decades since the first IFN treatment experience in mRCC (Quesada *et al*, 1983), the exact mechanisms underlying the antitumour response are not fully understood (Belardelli *et al*, 2002). Our tumour tissue analyses demonstrated only modest reduction in the number of proliferating tumour cells induced by IFN-*α* and IL-2, suggesting that immunotherapy reduces tumour size but has only limited effect on intrinsic tumour aggressiveness *in vivo*. We have previously demonstrated that localisation of CD4^+^, CD8^+^ and CD57^+^ lymphocytes to sites of tumour is a requisite for the response to therapy (Donskov *et al*, 2002a), thus hypothesising that the antitumour activity of IFN-*α in vivo* is primarily cellular immune mediated. This hypothesis is in accordance with previous *in vitro* findings (Kosmidis *et al*, 1992; Tsavaris *et al*, 1996).

In summary, the present study has assessed the Ki-67 (MIB-1) tumour proliferation marker in mRCC at baseline and during IL-2- and IFN-*α* based immunotherapy. Our data suggest that IFN-*α in vivo* has only modest effect on tumour proliferation in patients with mRCC. Tumour Ki-67 (MIB-1) reactivity after 1 month of immunotherapy appears to be a significant predictor of patient survival.
